# Evolution of Eschar in Scrub Typhus

**DOI:** 10.4269/ajtmh.16-0583

**Published:** 2016-12-07

**Authors:** Jin Park, Soo-Han Woo, Chang-Seop Lee

**Affiliations:** 1 Department of Dermatology, Chonbuk National University, Jeonju, Republic of Korea; 2 Department of Internal Medicine, Chonbuk National University, Jeonju, Republic of Korea; 3 Biomedical Research Institute of Chonbuk National University Hospital, Jeonju, Republic of Korea

A 60-year-old man presented with fever that developed 1 day before admission. The patient had recently returned from collecting acorns in the mountains 8 days before admission. Physical examination revealed a 0.5 × 0.5-cm eschar on the right chest wall. Indirect immunofluorescent antibody testing revealed an *Orientia tsutsugamushi* antibody titer of 1:2,560. The appearance of eschar was evaluated by dermoscopy and routine photography.

Figure 1A

**Figure 1.**
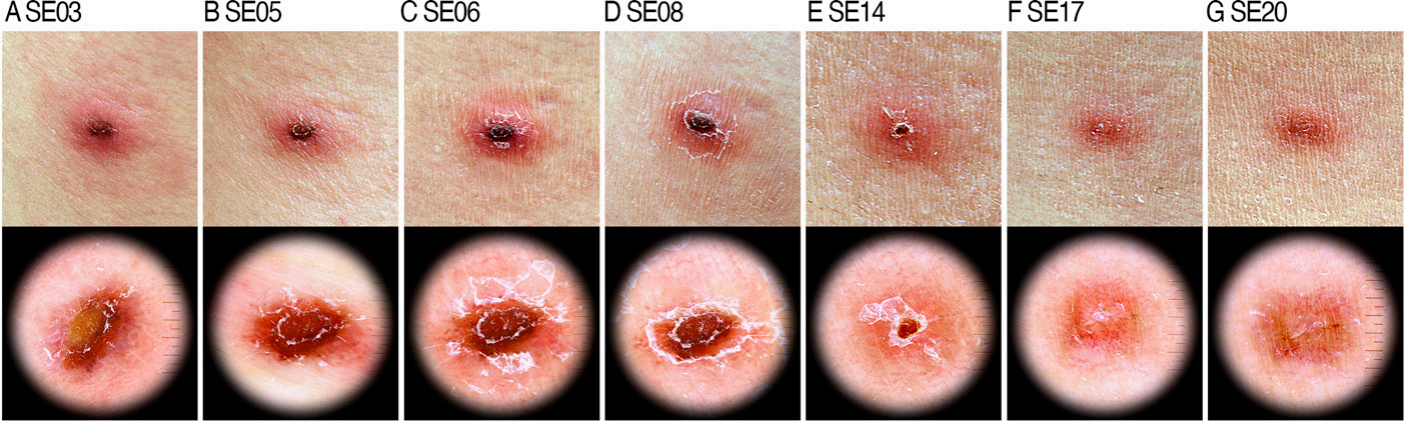
(**A**) Three days from symptom onset (SE03): central yellowish vesicle with mild whitish scale, and peripheral erythematous patch. (**B**) SE05: central vesicle turned into brown to black-colored crusts and scales are increased. (**C**) SE06: formation of typical eschar lesion having central black crusts and conspicuous erythematous patch with overlaying scale. (**D**) SE08: well-established eschar composed of three concentric components; innermost black crust outlined by inner scaly line, middle erythematous patch, and outermost whitish scaly layer. (**E**) SE14: shrinkage of central crusts and diminished peripheral scale. (**F**) SE17: central crust completely disappeared, and changed into central scar-like whitish area with peripheral erythematous area showing prominent vascular pattern. (**G**) SE20: dull reddish-brown hyperpigmentation.

–E clinical images show similar eschar morphology: a central black crust and peripheral erythematous rim visible to the naked eye. The visualization of subtle changes in eschar appearance can be improved by dermoscopy. In the early stages, a central vesicle surrounded by erythema can be observed, and whitish scales are seldom detected (Figure 1A; 3 days from symptom onset to eschar formation). With the progression of the lesion, a typical black crust appears in the center, and more apparent surrounding erythema can be seen (Figure 1B). The scales overlaying the crust gradually increase, and finally, the typical eschar is formed 6–8 days after onset (Figure 1C and D). After that, the crust slowly shrinks, and at the same time, the overlaying scales diminish (Figure 1E). After stage F, the crust disappears completely, leaving only a whitish scar-like macule (indicating fibrosis) (Figure 1F). The lesion heals with red-brown colored hyperpigmentation in place of surrounding erythema (Figure 1G). In the described case, the clinical course improved after 7 days of oral doxycycline treatment, and the patient was discharged.

Scrub typhus is an acute febrile illness caused by *O. tsutsugamushi*. Eschar is a necrotic lesion of the skin at the site of a chigger mite bite. The overall prevalence of eschar ranges widely in patients with scrub typhus.^1^ Eschar is a critical pathognomonic finding for clinical diagnosis of scrub typhus.^2^ The absence of eschar has been reported to be an independent predictive risk factor for fatal outcome.^3^ Therefore, the clinicians could early diagnose a scrub typhus if they are aware of the changing form of eschar over time, and would be able to promptly manage the patient with appropriate antibiotics.

## References

[1] Kim DM, Won KJ, Park CY, Yu KD, Kim HS, Yang TY, Lee JH, Kim HK, Song HJ, Lee SH, Shin H (2007). Distribution of eschars on the body of scrub typhus patients: a prospective study. Am J Trop Med Hyg.

[2] Shiao CC, Lin SY (2011). Eschar: a clue to scrub typhus. CMAJ.

[3] Lee CS, Hwang JH, Lee HB, Kwon KS (2009). Risk factors leading to fatal outcome in scrub typhus patients. Am J Trop Med Hyg.

